# Case Series: Video-Assisted Minimally Invasive Cardiac Surgery During Pregnancy

**DOI:** 10.3389/fmed.2021.781690

**Published:** 2021-12-22

**Authors:** Anyi Lu, Yingxian Ye, Jiaqi Hu, Ning Wei, Jinfeng Wei, Bimei Lin, Sheng Wang

**Affiliations:** ^1^Department of Anesthesiology, Guangdong Cardiovascular Institute & Guangdong Provincial People's Hospital, Guangdong Academy of Medical Sciences, Guangzhou, China; ^2^College of Medicine, Shantou University, Shantou, China; ^3^Department of Operation Room, Guangdong Cardiovascular Institute & Guangdong Provincial People's Hospital, Guangdong Academy of Medical Sciences, Guangzhou, China; ^4^Department of Anesthesiology, Linzhi People's Hospital, Linzhi, China

**Keywords:** minimally invasive cardiac surgery (MICS), video-assisted, pregnancy, cardiopulmonary bypass, perioperative management

## Abstract

Surgical intervention is expected to improve maternal outcomes in pregnant patients with heart disease once the conservative treatment fails. For pregnant patients with heart disease, the risk of cardiac surgery under cardiopulmonary bypass (CPB) must be balanced due to the high fetal loss. The video-assisted minimally invasive cardiac surgery (MICS) has been progressively applied and shows advantages in non-pregnant patients over the years. We present five cases of pregnant women who underwent a video-assisted minimally invasive surgical approach for cardiac surgery and the management strategies. In conclusion, the video-assisted MICS is feasible and safe to pregnant patients, with good maternal and fetal outcomes under the multidisciplinary assessment and management.

## Introduction

Heart diseases complicate 2–4% of pregnancies but account for up to 15% of maternal deaths (1). The cardiac potential to adapt hemodynamic change is impaired in women with structural heart disease, presented with reduced systolic and diastolic function (2). Once cardiac decompensation happens, cardiac surgery might be a solution for pregnant patients with structural heart diseases and compromised cardiac function. Maternal mortality after cardiac surgery during pregnancy is reported to be comparable to non-pregnant patients for about 11.2%, but the high fetal loss (33.1%) cannot be ignored (1). The management of these patients should be made with adequate multidisciplinary discussions, including cardiologists, anesthetists, and obstetricians, with aims to improve maternal and fetal outcomes.

The minimally invasive cardiac surgery (MICS) has been progressively applied in non-pregnant patients over the years and showed advantages, such as less transfusion rate and shorter postoperative ventilation support time as compared to that of mid-sternotomy approach thus, resulting to shorter ICU time and length of stay (3, 4). However, few MICS during pregnancy has been reported. This article presents a case series of five pregnant women who underwent a video-assisted MICS cardiac surgery during pregnancy in a tertiary medical center.

## Case Series

We retrospectively reviewed the records of all pregnancies with cardiac surgery in our hospital between 2019 and 2021. Only patients who underwent a video-assisted MICS (*n* = 5) were included. Informed consent has been obtained from all the patients. Baseline characteristics of all patients are shown in Table 1, and intraoperative and postoperative information of all patients are shown in Tables 2, 3.

**Table 1:** Baseline characteristics of five patients undergoing minimally invasive cardiac surgery (MICS) during pregnancy.

**Case no**.	**Age (years)**	**Gravida**	**Para**	**Weight** **(kg)**	**GA** **when cardiac surgery (weeks)**	**Diagnosis**	**NYHA grade**	**Preoperative transthoracic echocardiography**	**LVEF** **(%)**	**Pulmonary arterial systolic pressure (mmHg)**	**ECG**
1	35	5	2	60	18	Rheumatic heart disease	II	Severe mitral stenosis with moderate regurgitation, and mild tricuspid and aortic regurgitation	62	45	Normal
2	27	2	1	40	22	Infective endocarditis	II	Moderate mitral stenosis and severe mitral regurgitation with abnormal vegetation echo	60	80	Sinus tachycardia
3	38	2	0	56	18	Rheumatic heart disease	II	Moderate-severe mitral valve stenosis with moderate-severe regurgitation, moderate tricuspid regurgitation	73	62	Normal
4	34	2	1	58	31	Rheumatic heart disease	III	Severe mitral stenosis and mild mitral regurgitation	76	60	Normal
5	32	5	2	54	18	Left atrial myxoma	II	A medium-echo 19 mm × 10 mm irregular mass with good mobility in the left atrium, with good mobility and the stalk adherent to the fossa ovalis, considered as a myxoma	66	<40	Normal

*MICS, minimally invasive cardiac surgery; NYHA, New York Heart Association (NYHA) Classification; LVEF, left ventricular ejection fraction; ECG, electrocardiogram*.

**Table 2:** Intraoperative information of five patients undergoing MICS during pregnancy.

**Case no**.	**Intervention**	**Intubation**	**Total operation time (minutes)**	**CPB time (minutes)**	**Aortic cross-clamp time (minutes)**	**Lowest core temperature (°C)**	**Pulsatile perfusion**
1	Mitral valve replacement	Double lumen tube	145	75	47	36.0	Yes
2	Mitral valve replacement	Single lumen tube	165	92	64	36.0	Yes
3	Mitral valvuloplasty	Double lumen tube	170	88	68	36.0	Yes
4	Mitral valve replacement	Double lumen tube	133	67	43	36.0	Yes
5	Left atrial myxoma excision	Double lumen tube	135	46	21	35.7	No

**Table 3:** Postoperative information of five patients undergoing MICS during pregnancy.

**Case no**.	**Extubation time after surgery (hours)**	**Complication**	**Blood transfusion**	**Length of stay (days)**	**Maternal mortality**	**Gestational age when pregnancy termination (weeks)**	**Fetal outcomes**
1	7	No	No	11	No	20	Abortion
2	5	No	2U RBC	13	No	35	Abortion due to fetal cerebral anomaly
3	5	No	No	18	No	37	Normal Term Infant
4	10	Atrial fibrillation^*^	No	22	No	37	Normal Term Infant
5	1	No	2U RBC	13	No	26	Abortion due to fetal chromosomal abnormality

* *Four days after the surgery, the patient had an episode of acute atrial fibrillation with heart rate of 171 bpm. The sinus rhythm was returned with a heart rate of 92 bpm after the Valsava maneuver twice. One day after the first episode, the patients felt palpation with no reason and the ECG revealed a rapid onset of atrial fibrillation with a heart rate of 175 bpm. Antiarrhythmic drugs (12.5 mg beta-blocker and 0.2 mg deslanoside) were given and the episode was terminated. Beta-blocker was used to maintain the sinus rhythm*.

## Perioperative Management Strategy

Perioperative management was discussed by the multidisciplinary team that included cardiologists, cardiac surgeons, anesthesiologists, perfusionists, and gynecologists. The operative procedure was conducted under general anesthesia with 35 F left double-lumen intubation to allow single lung ventilation. A central venous catheter was placed on the right internal jugular vein. A 16 Fr venous cannula was placed in the superior cava vein through the right jugular vein for cardiopulmonary bypass (CPB) venous return. Transesophageal echocardiography was routinely set up for intraoperative monitoring. The patient was placed in a supine position with elevated right chest. Propofol and rocuronium were used for anesthetic induction. Sevoflurane, propofol, dexmedetomidine, and rocuronium were used for anesthetic maintenance with certain level of Nacrotrend values between 40 and 60. Sufentanil was intermittently given to ensure enough analgesia. Magnesium sulfate was used to inhibit uterine contraction. The fetal heart rate and uterine contraction were monitored by TEE and tocodynamometer. After heparinization, venous cannula and arterial cannula were placed in the right femoral vein and artery. A right anterolateral 4th intercostal 3.5 cm incision was made and the thoracoscopy was inserted via the 4th or 5th intercostal space. The high-flow, high-pressure normothermic CPB was then started. Vacuum-assisted venous drainage was also utilized (maximum negative pressure 20–40 mmHg). Cold Del Nido cardioplegia solution (blood and crystalloid mixed formula) was used as anterograde. During CPB, the hematocrit was maintained between 25 and 29%, as well as normothermia. Post-bypass transesophageal echocardiography has ensured a satisfying surgical outcome and fetal survival. An intercostal nerve block with 0.5% ropivacaine combined with intravenous analgesia, was used for postoperative multimodal analgesia.

The patient was transferred to the intensive care unit temporarily for monitoring. Fetal status was ensured by Doppler echography and uterine contraction was monitored by the tocodynamometer after the surgery. Atosiban was used postoperatively to inhibit uterine contraction. Warfarin and/or low molecular weight heparin were administrated for anticoagulation for those patients who underwent mechanical valve replacement.

## Discussion

### MICS During Pregnancy

Compared to standard sternotomy, the minimally invasive approach through thoracoscopy has been utilized in the past decades. However, there were few reports about occurrence of MICS with thoracoscopy during pregnancy. In one previous study, Nguyen et al. (5) reported a case of acute papillary muscle rupture during pregnancy. The minimally invasive mitral valve repair *via* the right thoracotomy was conducted, with main consideration on how morbidly obese this patient was. In our center, we conducted the MICS *via* video-assisted thoracoscopy, with the advantage of smaller operative incision, rather than the right thoracotomy. Also, favorable outcomes after the minimally invasive approach can be obtained more than that of standard sternotomy with less postoperative pain, faster recovery, less postoperative complication, and shorter length of stay in the hospital (6, 7). Qiu et al. (3) demonstrated that a full sternotomy was an independent risk factor for postoperative ventilation support. It was known that prolonged mechanical ventilation affects fetus morbidity and mortality in cardiac surgery during pregnancy (8). In our study, all fetuses remained alive after the cardiac surgery, supporting our supposition that the minimally invasive approach has its benefits to fetal survivals in pregnant women who underwent cardiac surgery under CPB. Also, sternal complications following a median sternotomy, including infection, sternal instability, and non-union, were reported by 1–8% worldwide (9). Sternal precautions were recommended for prevention of complication, which consisted of weight restrictions on the use of the upper limbs immediately after surgery for 6–12 weeks (10). This may interfere with normal maternal-infant bonding because motion restriction after median sternotomy may affect the mother in holding her child and in breastfeeding (5, 11). However, longer operation duration in the MICS should come into consideration for pregnant women as CPB time is reported as a risk factor for fetal mortality (12), but this may be solved by experienced surgeons. During MICS, single lung ventilation technique is required for a satisfactory field exposure. In our cases, we applied double lumen tube in four patients and single lumen tube in one patient. All surgical field remained satisfactory to the surgeons, including the one with single lumen intubation. Hypoxemia in the lung isolation after cardiopulmonary bypass (CPB) surgery might impair fetal oxygenation, and whether single lumen tube in MICS benefits to these patients still needs further investigation.

### Cardiopulmonary Bypass During Pregnancy

Cardiopulmonary bypass can pose significant effects on both the fetus and the mother. Sustained uterine contraction during CPB is regarded as a risk factor to fetus survival. The cooling and rewarming process during CPB induces uterine contraction, especially after maternal hypothermia, which induces placental hypoperfusion and, consequently, fetal hypoxia. Hemodilution of progesterone during CPB also enhances uterine contraction (12, 13). In our study, we performed high perfusion pressure and normothermic CPB to ensure placental perfusion. It is thought that pulsatile perfusion can release endothelium-derived growth factors from the vascular endothelium and reduce uterine contractions, which may result to good fetal outcomes in pregnant women who undergo cardiac surgery (12, 14). In our study, non-pulsatile CPB was performed in two cases and both fetuses were alive after cardiac surgery, though 1 patient eventually has terminated pregnancy due to fetal chromosomal abnormality. Pulsatile perfusion was performed in three patients and one patient has terminated pregnancy due to fetal cerebral anomaly. In fact, there are few clinical data to support the advantage of pulsatile perfusion over non-pulsatile perfusion in pregnant women. In the cohort study of John et al. (15), there was a reported three fetal deaths among the 21 non-pulsatile CPB cases. Most of them (two fetal deaths) happened in women with other comorbidities. Further clinical research evidence is required to determine the beneficial application of pulsatile or non-pulsatile perfusion in cardiac surgery of pregnant women.

In the study of Jha et al. (1), the pooled rate of maternal complications was at 15%, maternal heart failure at 5.8%, and arrhythmia at 2.1%, respectively. In our study, only 1 patient experienced cardiovascular complication of acute onset of atrial fibrillation that requires treatment. Maternal mortality is comparable to that of CPB in non-pregnant women in the previous studies, with the estimated rate of 11% in the meta-analysis of Jha et al. (1). Maternal status with worse NYHA and emergency surgery contributed to unfavorable maternal outcomes in these patients. In our cases, all women survived and may benefit from good maternal NYHA status and semi-urgent surgery.

### Management of Cardiac Surgery During Pregnancy

The decision to perform cardiac surgery during pregnancy should be thoroughly discussed within a multidisciplinary team of obstetricians, cardiologists, cardiac surgeons, anesthetists, and gynecologists. The Modified World Health Organization Classification of Cardiovascular Disease in Pregnancy is used as reference for risk stratification of maternal and neonatal complication (16). Compared to non-pregnant women, pregnant women are at higher risk of aspiration, difficult intubation, and thromboembolism, which made them require more attention in preoperative preparation (17). Once the patient is supine, the 15° position of left uterine displacement should be applied to avoid aortocaval compression after 18–20 weeks of gestation (18).

Pregnant patients are more sensitive to IV and inhalational medications. Propofol seems to be the preferred medication for induction in healthy pregnant patients. Mongardon et al. (19) demonstrated that the dose of propofol required in pregnant women for loss of consciousness is 8% less than in non-pregnant patients. Inhalation medication, such as desflurane and sevoflurane, inhibits myometrial contractions during the operation, which may be beneficial to pregnant women undergoing cardiac surgery (20). It suggested a more rapid onset of neuromuscular block with vecuronium and rocuronium in pregnant women (18).

Sympathomimetic agents, such as phenylephrine and norepinephrine, are safe to maintain blood pressure. In comparison with phenylephrine, ephedrine may act on fetal metabolism and be associated with neonatal acidosis and, therefore, should be considered as secondary choice of vasopressor in pregnancy (16, 21).

**Figure 1:**
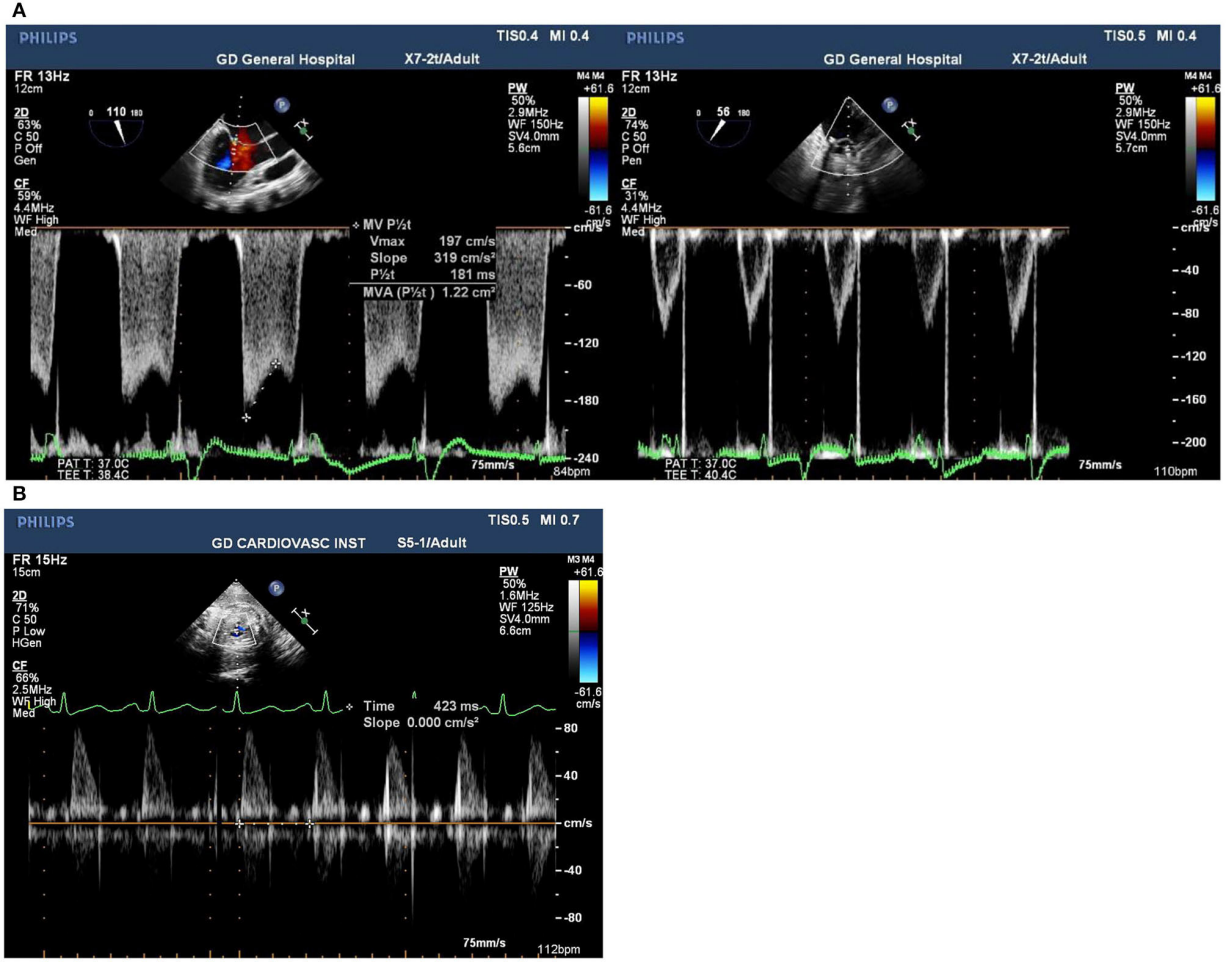
**(A)** Pre and post-surgical mitral valve view of transesophageal echocardiogram images of mitral valve stenosis in Case 1. **(B)** Postoperative transesophageal echocardiogram images of Doppler of fetal blood flow in Case 3 presenting the fetal heart rate at 141 bpm.

Intraoperative monitoring for both the mother and the fetus is critically significant to favorable maternal and fetal outcomes. If uterine contractions are detected, increase maternal intravascular volume may be helpful and tocolytic treatment can be administrated (22). Fetal bradycardia is an important indicator of fetal distress during CPB, which usually occurs at the initiation of CPB caused by a decrease in systemic vascular resistance, thereby affected by hemodilution, and the release of vasoactive substances. It has been reported that fetal heart rate (FHR) monitoring with an external cardiotocography reduces fetal mortality to 7.5% in cardiac surgeries with CPB (23). In our cases, the tocodynamometer combined with TEE were used to monitor uterine contraction and fetal heart rate (Figure 1). FHR was measured intraoperatively by Doppler echocardiography across the fetal blood flow, which was available in all pregnant women in our cases whose gestational age ranged from 18 to 31 weeks.

Commonly tocolytic drugs include magnesium, beta-adrenergic drugs, nitroglycerin, and prostaglandin inhibitors (22). In our case, we chose magnesium sulfate, which is mainly used for pre-eclampsia control, to inhibit uterine contraction by decreasing acetylcholine transmission in motor nerve terminals (24). Also, it was reported that antenatal usage of magnesium sulfate may contribute to fetal neuroprotection and may reduce the risk of cerebral palsy or even death (25). However, magnesium may potentiate the activity of both depolarizing and non-depolarizing neuromuscular blocking agents (NMBA). Consequently, the dose of NMBA should be reduced (24). In a case of left atrial myxoma resection reported by Alexis et al. (26), a low dose of nicardipine, a calcium channel blocker, was used to inhibit uterine contractility and may show an advantage to restore FHR.

For these patients, postoperative monitoring is pivotal along with the assessment of fetus by using Doppler ultrasound, as well checking of uterine contraction with a tocodynamometer. If necessary, tocolytic drugs should be administrated in case of a preterm labor. The left lateral position should be maintained to prevent aortocaval compression (18). Furthermore, postoperative analgesia is important for pain control and can reduce the risk of premature labor. NSAIDs should be avoided in women as prenatal exposure to NSAIDs after 30 weeks gestational age is associated with an increased risk of premature closure of the fetal ductus arteriosus and oligohydramnios (27). In our center, multimodal analgesia was performed in every patient including intercostal nerve block with 0.5% ropivacaine and intravenous analgesia with opioids, resulting all patients to report a pain score <3 on a numeric rating scale (NRS).

## Conclusion

The video-assisted MICS is feasible and safe with good maternal and fetal outcomes, which may be progressively applied in patients in the need for cardiac surgery during pregnancy. The multidisciplinary team for decision in the management of these patients is of vital importance to favorable outcome for both the mother and the fetus.

## Data Availability Statement

The raw data supporting the conclusions of this article will be made available by the authors, without undue reservation.

## Ethics Statement

The studies involving human participants were reviewed and approved by Guangdong Provincial People's Hospital Ethics Committee. The patients/participants provided their written informed consent to participate in this study. Written informed consent was obtained from the individual(s) for the publication of any potentially identifiable images or data included in this article.

## Author Contributions

NW, YY, and JH collected and organized the information of patients. AL wrote the first draft of the manuscript. JW, YY, JH, BL, and NW wrote sections of the manuscript. All authors contributed to manuscript revision, read, and approved the submitted version.

## Funding

This grant of the study was from the Natural Science Foundation of Tibet Autonomous Region [XZ2020ZR-ZY55(Z)].

## Conflict of Interest

The authors declare that the research was conducted in the absence of any commercial or financial relationships that could be construed as a potential conflict of interest.

## Publisher's Note

All claims expressed in this article are solely those of the authors and do not necessarily represent those of their affiliated organizations, or those of the publisher, the editors and the reviewers. Any product that may be evaluated in this article, or claim that may be made by its manufacturer, is not guaranteed or endorsed by the publisher.
