# Corrigendum: Case series: Video-assisted minimally invasive cardiac surgery during pregnancy

**DOI:** 10.3389/fmed.2022.1054415

**Published:** 2022-10-28

**Authors:** Anyi Lu, Yingxian Ye, Jiaqi Hu, Ning Wei, Jinfeng Wei, Bimei Lin, Sheng Wang

**Affiliations:** ^1^Department of Anesthesiology, Guangdong Cardiovascular Institute & Guangdong Provincial People's Hospital, Guangdong Academy of Medical Sciences, Guangzhou, China; ^2^College of Medicine, Shantou University, Shantou, China; ^3^Department of Operation Room, Guangdong Cardiovascular Institute & Guangdong Provincial People's Hospital, Guangdong Academy of Medical Sciences, Guangzhou, China; ^4^Department of Anesthesiology, Linzhi People's Hospital, Linzhi, China

**Keywords:** minimally invasive cardiac surgery (MICS), video-assisted, pregnancy, cardiopulmonary bypass, perioperative management

In the published article, there was an error in Table 2 as published. The correct data should be yes for the pulsatile perfusion in case 4. The corrected and its caption appear below.

**Table 2:** Intraoperative information of five patients undergoing MICS during pregnancy.

**Case no**.	**Intervention**	**Intubation**	**Total operation time (minutes)**	**CPB time (minutes)**	**Aortic cross-clamp time (minutes)**	**Lowest core temperature (°C)**	**Pulsatile perfusion**
1	Mitral valve replacement	Double lumen tube	145	75	47	36.0	Yes
2	Mitral valve replacement	Single lumen tube	165	92	64	36.0	Yes
3	Mitral valvuloplasty	Double lumen tube	170	88	68	36.0	Yes
4	Mitral valve replacement	Double lumen tube	133	67	43	36.0	Yes
5	Left atrial myxoma excision	Double lumen tube	135	46	21	35.7	No

In the published article, there was an error in Table 3 as published. The Extubation time after surgery in Case no. 1 should be “7” h, and the Blood transfusion for Case no. 2 should be “2U blood transfusion.” The corrected Table 3 and its caption appear below.

**Table 3:** Postoperative information of five patients undergoing MICS during pregnancy.

**Case no**.	**Extubation time after surgery (hours)**	**Complication**	**Blood transfusion**	**Length of stay (days)**	**Maternal mortality**	**Gestational age when pregnancy termination (weeks)**	**Fetal outcomes**
1	7	No	No	11	No	20	Abortion
2	5	No	2U RBC	13	No	35	Abortion due to fetal cerebral anomaly
3	5	No	No	18	No	37	Normal Term Infant
4	10	Atrial fibrillation^*^	No	22	No	37	Normal Term Infant
5	1	No	2U RBC	13	No	26	Abortion due to fetal chromosomal abnormality

* Four days after the surgery, the patient had an episode of acute atrial fibrillation with heart rate of 171 bpm. The sinus rhythm was returned with a heart rate of 92 bpm after the Valsava maneuver twice. One day after the first episode, the patients felt palpation with no reason and the ECG revealed a rapid onset of atrial fibrillation with a heart rate of 175 bpm. Antiarrhythmic drugs (12.5 mg beta-blocker and 0.2 mg deslanoside) were given and the episode was terminated. Beta-blocker was used to maintain the sinus rhythm.

In the published article, there was an error. A correction has been made to **Perioperative management strategy**, Paragraph 1. This sentence previously stated:

“Sevoflurane, propofol, dexmedetomidine, and rocuronium were used for anesthetic maintenance with certain level of Nacrotrend Bis values between 40 and 60.”

The corrected sentence appears below:

“Sevoflurane, propofol, dexmedetomidine, and rocuronium were used for anesthetic maintenance with certain level of Nacrotrend values between 40 and 60.”

In the published article, there was an error.

A correction has been made to **Perioperative management strategy**, Paragraph 1. This sentence previously stated:

“After heparinization, a 24 Fr venous cannula and 19 Fr arterial cannula were placed in the right femoral vein and artery.”

The corrected sentence appears below:

“After heparinization, venous cannula and arterial cannula were placed in the right femoral vein and artery.”

In the published article, there was an error. A correction has been made to **Perioperative management strategy**, Paragraph 1. This sentence previously stated:

“the thoracoscopy was inserted *via* the 5th intercostal space.”

The corrected sentence appears below:

“the thoracoscopy was inserted *via* the 4th or 5th intercostal space.”

The authors apologize for these errors and state that this does not change the scientific conclusions of the article in any way. The original article has been updated.

## Publisher's note

All claims expressed in this article are solely those of the authors and do not necessarily represent those of their affiliated organizations, or those of the publisher, the editors and the reviewers. Any product that may be evaluated in this article, or claim that may be made by its manufacturer, is not guaranteed or endorsed by the publisher.

